# Supplementary material for: The Absence of Bovine Serum Albumin (BSA) in Preimplantation Culture Media Impairs Embryonic Development and Induces Metabolic Alterations in Mouse Offspring
Source: Int J Mol Sci. 2025 Jul 21;26(14):6989. doi: 10.3390/ijms26146989 (PMC12295788; doi:10.3390/ijms26146989)
Supplement: Supplementary file 1 [file ijms-26-06989-s001.zip › ijms-3713100-supplementary.pptx]

## Slide 1
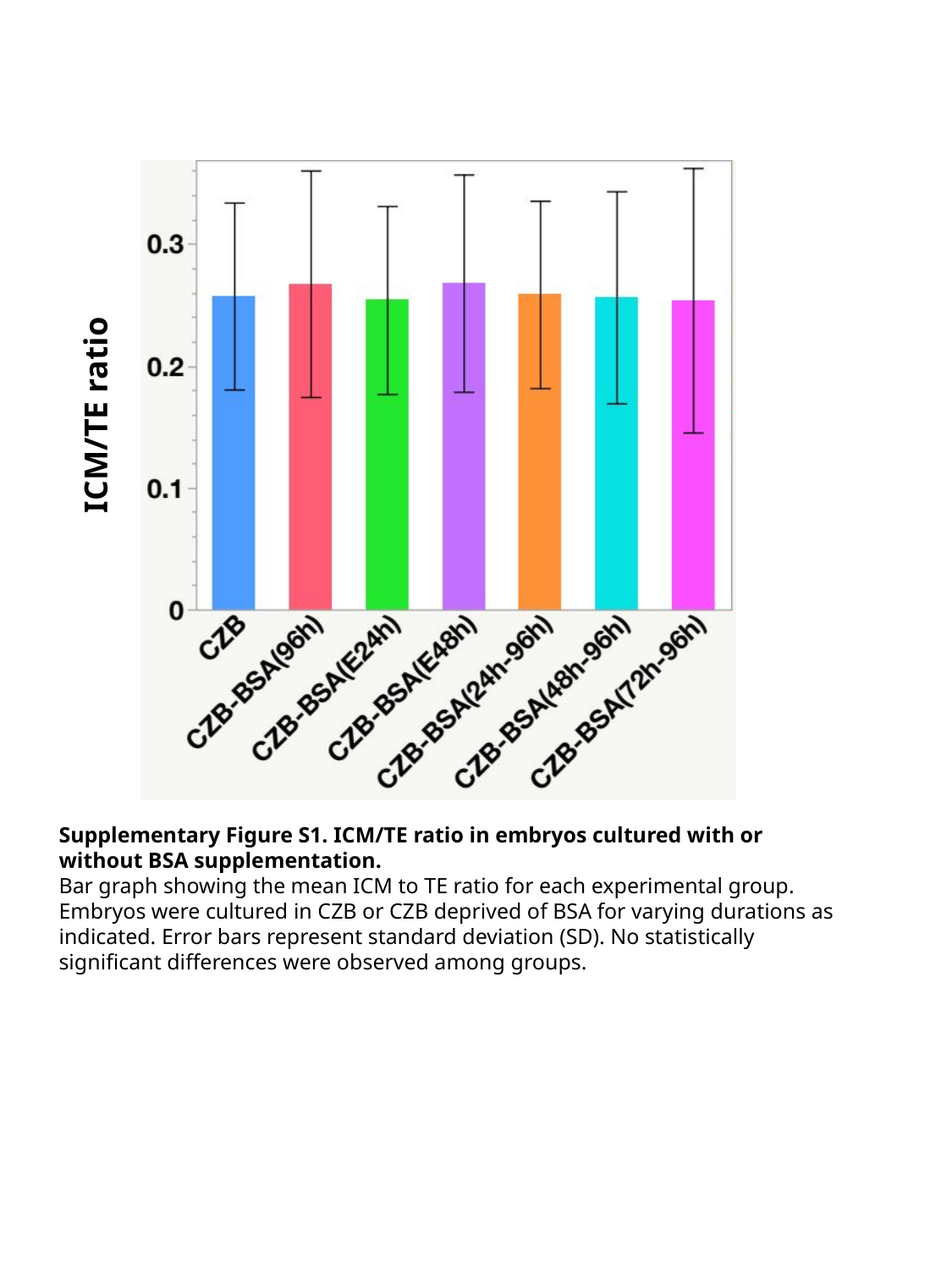

ICM/TE ratio
Supplementary Figure S1. ICM/TE ratio in embryos cultured with or without BSA supplementation.
Bar graph showing the mean ICM to TE ratio for each experimental group. Embryos were cultured in CZB or CZB deprived of BSA for varying durations as indicated. Error bars represent standard deviation (SD). No statistically significant differences were observed among groups.
